# Simultaneous detection of seven bacterial pathogens transmitted by flies using the reverse line blot hybridization assay

**DOI:** 10.1186/s13071-024-06170-3

**Published:** 2024-02-22

**Authors:** Yonghua Ma, Qingli Niu, Xiaolin Sun, Yuanyuan Li, Huitian Gou, Zexiang Wang, Beibei Song

**Affiliations:** 1 College of Veterinary Medicine, Gansu Agricultural University, Lanzhou, China; 2 Lanzhou Veterinary Research Institute, Chinese Academy of Agricultural Science, Lanzhou, China

**Keywords:** Reverse line blot, Fly-borne, Hybridization assay, Bacterial pathogens, Probes

## Abstract

**Background:**

Traditional methods for detecting insect-borne bacterial pathogens are time-consuming and require specialized laboratory facilities, limiting their applicability in areas without access to such resources. Consequently, rapid and efficient detection methods for insect-borne bacterial diseases have become a pressing need in disease prevention and control.

**Methods:**

We aligned the ribosomal 16S rRNA sequences of seven bacterial species (*Staphylococcus aureus, Shigella flexneri, Aeromonas caviae, Vibrio vulnificus, Salmonella enterica, Proteus vulgaris*, and *Yersinia enterocolitica*) by DNASTAR Lasergene software. Using DNASTAR Lasergene and Primer Premier software, we designed universal primers RLB-F and RLB-R, two species-specific probes for each pathogen, and a universal probe (catch-all). The PCR products of seven standard strains were hybridized with specific oligonucleotide probes fixed on the membrane for specific experimental procedures. To evaluate the sensitivity of PCR-RLB, genomic DNA was serially diluted from an initial copy number of 10^10^ to 10^0^ copies/μl in distilled water. These dilutions were utilized as templates for the PCR-RLB sensitivity analysis. Simultaneous detection of seven fly-borne bacterial pathogens from field samples by the established PCR-RLB method was conducted on a total of 1060 houseflies, collected from various environments in Lanzhou, China.

**Results:**

The established PCR-RLB assay is capable of detecting bacterial strains of about 10^3^ copies/μl for *S. aureus*, 10^3^ copies/μl for *S. flexneri*, 10^5^ copies/μl for *A. caviae*, 10^5^ copies/μl for *V. vulnificus*, 10^0^ copies/μl for *S. enterica*, 10^5^ copies/μl for *P. vulgaris*, and 10^0^ copies/μl for *Y. enterocolitica.* The results demonstrate that the detection rate of the established PCR-RLB method is higher (approximately 100 times) compared to conventional PCR. This method was applied to assess the bacterial carrier status of flies in various environments in Lanzhou, China. Among the seven bacterial pathogens carried by flies, *S. enterica* (34.57%), *S. flexneri* (32.1%), and *Y. enterocolitica* (20.37%) were found to be the predominant species.

**Conclusions:**

Overall, this research shows that the rapid and efficient PCR-RLB detection technology could be a useful for surveillance and therefore effective prevention and control the spread of insect-borne diseases. Meanwhile, the experimental results indicate that urban sanitation and vector transmission sources are important influencing factors for pathogen transmission.

**Graphical Abstract:**

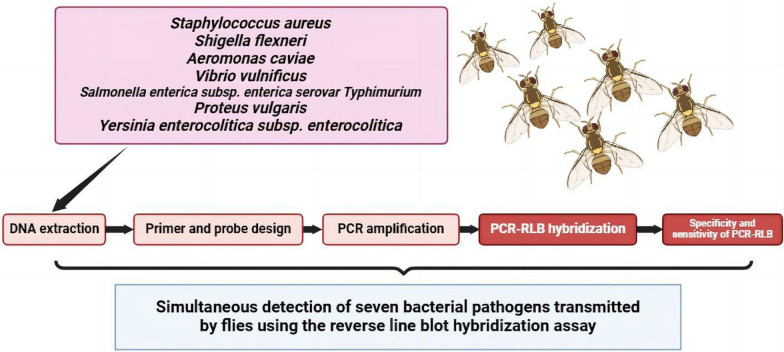

## Background

Flies are known carriers and disseminators of various bacterial pathogens, which can lead to human diarrhea, food poisoning, and a range of bacterial diseases, including cholera, bacteremia, tuberculosis, and anthrax [1–7]. While numerous fly species exist, only a few are commonly found in households and farms, such as the houseflies *Stomoxys calcitrans*, *Lucilia sericata*, and *Sarcophagidae*. Flies often feed and reproduce in animal feces, organic waste, and carcasses, making them significant contributors to environmental contamination and human health risks [4, 8–14]. Notably, flies have been implicated in the transmission of enterohemorrhagic *Escherichia coli* and avian influenza in Japan, as reported in the Science Times on February 24, 2005. In China, flies are also a key focus of prevention and control measures for insect-borne diseases.

Traditionally, the detection of insect-borne bacterial pathogens relies on labor-intensive bacterial culture and isolation methods. This approach is time-consuming and requires specialized laboratory facilities, limiting its applicability in areas without access to such resources. Consequently, rapid and efficient detection methods for insect-borne bacterial diseases have become a pressing need in disease prevention and control.

Advancements in bioinformatics have yielded complete sequences of various insect-borne pathogens, offering valuable tools for pathogen gene detection. Molecular biology has also led to the development of efficient and specific techniques for detecting vector-borne diseases, including polymerase chain reaction-enzyme linked immunosorbent assay (PCR-ELISA) [15], real-time PCR (qPCR) [16], and nucleic acid probe hybridization. Among these techniques, PCR-based reverse line blot (PCR-RLB) stands out for its ability to simultaneously detect a wide range of pathogenic microorganisms carried by insects, offering high sensitivity, specificity, and throughput. This method involves combining single-stranded PCR products with species-specific probes to identify the amplified sequence, making it suitable for species identification and differentiation in cases of mixed infections. PCR-RLB has found extensive application in the detection of various pathogens. For instance, Kaufhold et al. [17] initially employed PCR-RLB for serotype identification of *Streptococcus*, followed by O'Sullivan et al. [18], who analyzed drug-resistant strains of *Staphylococcus aureus* using PCR-RLB technology. Nijhof et al. [19] also utilized this method to analyze three species of *Theileria* in Africa. However, most previous studies have primarily focused on detecting single bacterial subtypes, with limited reports on the simultaneous detection of multiple bacterial species.

Between 2004 and 2010, enteric diseases, including bacillary dysentery, typhoid, and hepatitis A, accounted for a significant portion (0.24–0.44) of the total incidence of Category A, B, and C infectious diseases in Lanzhou, China. In recent years, there has been a notable increase in the incidence of intestinal infectious diseases and several reported cases of bacterial food poisoning. This underscores the ongoing significance of intestinal infectious diseases and bacterial food poisoning in Lanzhou's disease prevention and control efforts. Therefore, the development of a rapid method for detecting intestinal infectious bacteria carried by flies is a crucial step in preventing and controlling infectious diseases. The primary objective of this study was to establish a PCR-RLB hybridization assay capable of simultaneously detecting seven bacterial species, namely *S. aureus*, *Shigella flexneri*, *Aeromonas caviae*, *Vibrio vulnificus*, *Salmonella enterica subsp. enterica serovar Typhimurium*, *Proteus vulgaris*, and *Yersinia enterocolitica subsp. enterocolitica*, in a single reaction system. This method was then applied to assess bacterial carriage by houseflies randomly collected from four different environments in Lanzhou, China, including residential areas, slaughterhouses, garbage sites, and hospitals.

## Methods

### Standard bacterial strains

We obtained seven bacterial strains from Shanghai Bioplus Biotech Co., Ltd. (Shanghai, China), and their details are provided in Table 1. These strains underwent identification using the VITEK 2 Compact automatic bacterial identification and analysis system (Meriere, France), conducted at the microbiology laboratory of the Quarantine Service, Gansu Provincial Center for Disease Control and Prevention (GSCDC) in Lanzhou, Gansu Province, China.

**Table 1:** Standard strains of seven bacterial species

Species	Strain ID number
*Staphylococcus aureus*	ATCC 25923
*Shigella flexneri*	ATCC 12022
*Aeromonas caviae*	ATCC 15468
*Vibrio vulnificus*	ATCC 17802
*Salmonella enterica subsp. enteric serovar typhimurium*	ATCC 13311
*Proteus vulgaris*	ATCC 29905
*Yersinia enterocolitica subsp. Enterocolitica*	ATCC 17802

ATCC, American Type Culture Collection

### Collection and treatment of housefly samples

We randomly collected a total of 1060 houseflies from various locations in Lanzhou, China, between 2016 and 2017. These locations included residential areas (*n* = 380), slaughterhouses (*n* = 330), garbage transfer stations (*n* = 200), and areas near hospitals (*n* = 150). The samples were processed in accordance with industry standard SN/T 3064.1–2011. Specifically, 10 houseflies were grouped together in sterilized triangular flasks, and 10 ml of physiological saline was added. The mixture was shaken for 10 min by orbital shaker (Orbital Shaker TS-1) to prepare for subsequent DNA extraction.

### DNA extraction

We extracted DNA from fly samples and standard bacterial strains using gram-negative bacterial DNA extraction kit (ABT, Beijing, China), following the manufacturer's instructions. In summary, 1 ml of the overnight bacterial culture or homogenized fly sample was collected and centrifuged for 5 min at 10,000 rpm. The supernatant was discarded, and 1 ml of physiological saline was added to the precipitate. After agitation to disperse the bacteria, the mixture was centrifuged for 5 min at 10,000 rpm. The supernatant was once again discarded, and approximately 200 μl of sterilized ddH_2_O was added and mixed thoroughly. After another centrifugation step for 3 min at 13,000 rpm, the supernatant was discarded. Subsequently, 50 μl of the nucleic acid extraction solution from the gram-negative bacterial DNA extraction kit was added to the bacterial precipitate, mixed thoroughly, and centrifuged briefly. The supernatant was collected, and the bacterial solution in the EP (Eppendorf) tube was subjected to a water bath at 100 °C for 10 min. Following this, it was centrifuged for 10 min at 13,000 rpm, and the resulting supernatant was stored at − 20 °C for subsequent use as the DNA template in amplification experiments.

### Primer and probe design

The 16S RNA gene sequences are highly conserved and are available in the GenBank database [20]. We aligned the ribosomal 16S rRNA sequences of seven bacterial species (*S. aureus, S. flexneri, A. caviae, V. vulnificus, S. enterica, P. vulgaris*, and *Y. enterocolitica*) by DNASTAR Lasergene software (DNASTAR, Inc, USA). Using DNASTAR Lasergene and Primer Premier software (PREMIER Biosoft, USA), we designed universal primers RLB-F and RLB-R, two species-specific probes for each pathogen, and a universal probe (catch-all). The theoretical specificity of all primers and probes was validated through alignment and verification against the National Center for Biotechnology Information (NCBI) sequence databases using the Basic Local Alignment Search Tool (BLASTn). Universal primers were biotin-labeled at the 5’-end to detect PCR products in PCR RLB assays through hybridization with streptavidin-peroxidase substrates. All probes were labeled with an amine group at the 5’-end to facilitate covalent bonding with nylon membranes, enabling membranes to be stripped and reused. The primers and probes were synthesized by Sangon Biotech Company, China (Tables 2, 3).

**Table 2:** Primer sequences and concentrations

Primer	Primer sequence (5’–3’)	Total provision (O.D.)	Optimal concentrations (μM)	Length
RLB-F	AGYGGCGGACGGGTGAGTAA	5	50	1100 bp
RLB-R	Biotin-CCATTGTAGCACGTGTGTAGCCC	5	50

**Table 3:** Probe sequences and concentrations

Probe	Probe sequence (5’–3’)	Accession no.	Base number	Total provision (O.D.)	Optimal concentrations (μM)	Position
Catch-all	(NH_2_)-CAGGATTAGATACCCTGGTAGTCC	–	24	10	50	820–843 bp
*Staphylococcus aureus*-1	(NH_2_)-TCAAAAGTGAAAGACGGTCTTGC	NR118997	23	10	–	220–242 bp
*Staphylococcus aureus-*2	(NH_2_)-CAACATATGTGTAAGTAACTGTGCAC	NR118997	26	10	50	480–505 bp
*Shigella flexneri*-1	(NH_2_)-GGAGTAAAGTTAATACCTTTGC	X96963	22	10	–	480–501 bp
*Shigella flexneri*-2	(NH_2_)-CTGATACTGGCAAGCTTGAGTCTCGT	X96963	26	10	50	670–695 bp
*Aeromonas caviae*-1	(NH_2_)-CGAGGAGGAAAGGTCAGTAGC	NR029252	21	10	–	108–128 bp
*Aeromonas caviae*-2	(NH_2_)-GGAATCAGAACACAGGTGCT	NR029252	20	10	100	698–717 bp
*Vibrio vulnificus*	(NH_2_)-AGAGAATTCTAGCGGAGACGCG	NR118930	22	10	100	665–686 bp
*Salmonella enterica*	(NH_2_)-AGAAGAATCCAGAGATGGATTG	NR119108	22	10	100	666–687 bp
*Proteus vulgaris-*1	(NH_2_)-GGTGATAAAGTTAATACCTTTGTCAA	NR115878	26	10	100	118–143 bp
*Proteus vulgaris*-2	(NH_2_)-CGAATCCTTTAGAGATAGAGGA	NR115878	22	10	–	667–688 bp
*Yersinia enterocolitica*-1	(NH_2_)-GGCCAATAACTTAATAGGTTG	NR074308	21	10	–	118–138 bp
*Yersinia enterocolitica*-2	(NH_2_)-AGAACTTAGCAGAGATGCTTCG	NR074308	22	10	100	667–688 bp

Two probes were designed for each pathogen species for specific probe screening

### PCR amplification

For each sample, the reaction mixture was prepared as follows:

**Table:** 

Template DNA	1 µl
10 × reaction buffer*	10.0 μl
10 mM dNTP	1.0 μl
20 μM sense primer (RLB F)	0.5 μl
20 μM antisense primer (RLB R)	0.5 μl
Taq polymerase	0.5 μl
H_2_O	11.5 μl
Total	25 μl
*(200 mM Tris–HCl(pH 8.55), 160 mM (NH_4_)_2_SO_4_, and 20 mM MgCl_2_)	

Genomic DNA from either the standard strains or the samples was used as template for the PCR reactions. The PCR reaction commenced with an initial denaturation step at 94 °C for 5 min. This was followed by 35 cycles consisting of denaturation at 94 °C for 30 s, annealing at 63 °C for 30 s, and extension at 72 °C for 45 s. A final extension step at 72 °C for 10 min concluded the PCR process. Subsequently, the samples were maintained at 12 °C until analysis; 1 μl of genomic DNA from standard bacterial strains was added for the positive control. For negative control, nothing was added. The PCR amplification products were subjected to electrophoresis using a 1% agarose gel. Gels were stained with Goldview nucleic acid gel stain and visualized under ultraviolet (UV) light.

### PCR-RLB hybridization

The PCR-RLB protocol was executed following established procedures [21]. In summary, a Biodyne C membrane (BNBCH5R, Pall BioSupport) was activated at 25 °C by immersing it in 16% EDAC (E7750, Sigma) for 10 min. Subsequently, it was rinsed with distilled water and placed in a blot processor (Miniblotter, US Patent). Species-specific oligonucleotide probes were diluted to various concentrations (25, 50, 100, 200, 500, 800, and 1000 μM) in 500 mM NaHCO_3_ (pH 8.4). These diluted probes were then added to the slots of the blot processor and allowed to incubate for 2 min. The membrane was subsequently immersed in 100 mM NaOH for 10 min and rinsed with demineralized water at 60 °C for 5 min in 2 × SSPE/0.1% SDS. The membrane was then positioned perpendicular to the probe orientation in the blot processor.

Next, 20 μl of each PCR product of the sample was diluted in 2 × SSPE with 10% w/v SDS to a final volume of 150 μl. This mixture was heated to 99 °C for 10 min and promptly cooled on ice. The denatured PCR products were added to the slots in the blot processor and incubated for 60 min at 60 °C. Subsequently, the membrane was washed twice at 60 °C for 10 min in 2 × SSPE with 0.5% SDS. Furthermore, the membrane was treated at 42 °C for 60 min with peroxidase-labeled streptavidin, which was diluted 1:4000 in 2 × SSPE/0.5% SDS. It was then washed twice at 42 °C for 10 min in 2 × SSPE/0.5% SDS and twice at room temperature for 5 min in 2 × SSPE. Finally, chemiluminescence detection was carried out in accordance with standard procedures (Amersham).

### Specificity and sensitivity of PCR-RLB

Two specific oligonucleotide probes were designed for each pathogen. The standard strains were amplified using a pair of universal primers. The PCR products of seven standard strains were hybridized with specific oligonucleotide probes fixed on the membrane for specific experimental procedures (the specific operation is as shown in 2.6). The specificity of the probe can be confirmed by binding with PCR product of their corresponding standard strain and not hybridizing with the blank control and that of other strain.

To evaluate the sensitivity of PCR-RLB, the concentrations of genomic DNA from the standard strains were measured using a nucleic acid concentration meter (NanoDrop ND-2000). DNA copy number was then calculated by the following formula:

$$Copies/\mu L=\frac{\mathrm{DNA\;concentration }\;\left(ng/\mu {\text{L}}\right)\times {10}^{-9}\times 6.02\times {10}^{23}}{\mathrm{DNA\; length}\times 330}$$

Genomic DNA was serially diluted from an initial copy number of 10^10^ to 10^0^ copies/μl in distilled water. These dilutions were utilized as templates for the PCR-RLB sensitivity analysis (the experimental method is detailed in 2.6).

## Results

### Selection of probes and primers

**Fig. 1:**
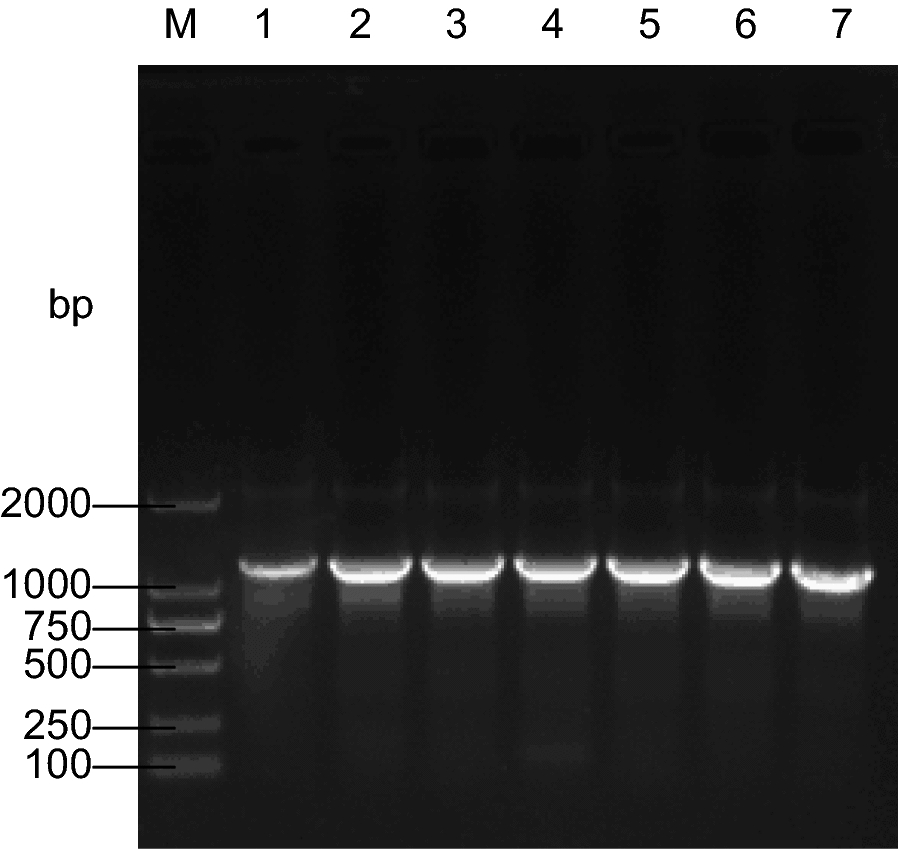
Validation results of primers. Lane: M, DL2000 DNA marker; lane 1–7: *Staphylococcus aureus*, *Shigella flexneri*, *Aeromonas caviae*, *Vibrio vulnificus*, *Salmonella enterica*, *Proteus vulgaris*, and *Yersinia enterocolitica*, respectively

A pair of primers (RLB-F/R) with lengths of 20 and 23 bp were designed to amplify all standard strains, and the amplicon size was approximately 1100 bp. The results of the PCR amplification are illustrated in Fig. 1.

**Fig. 2:**
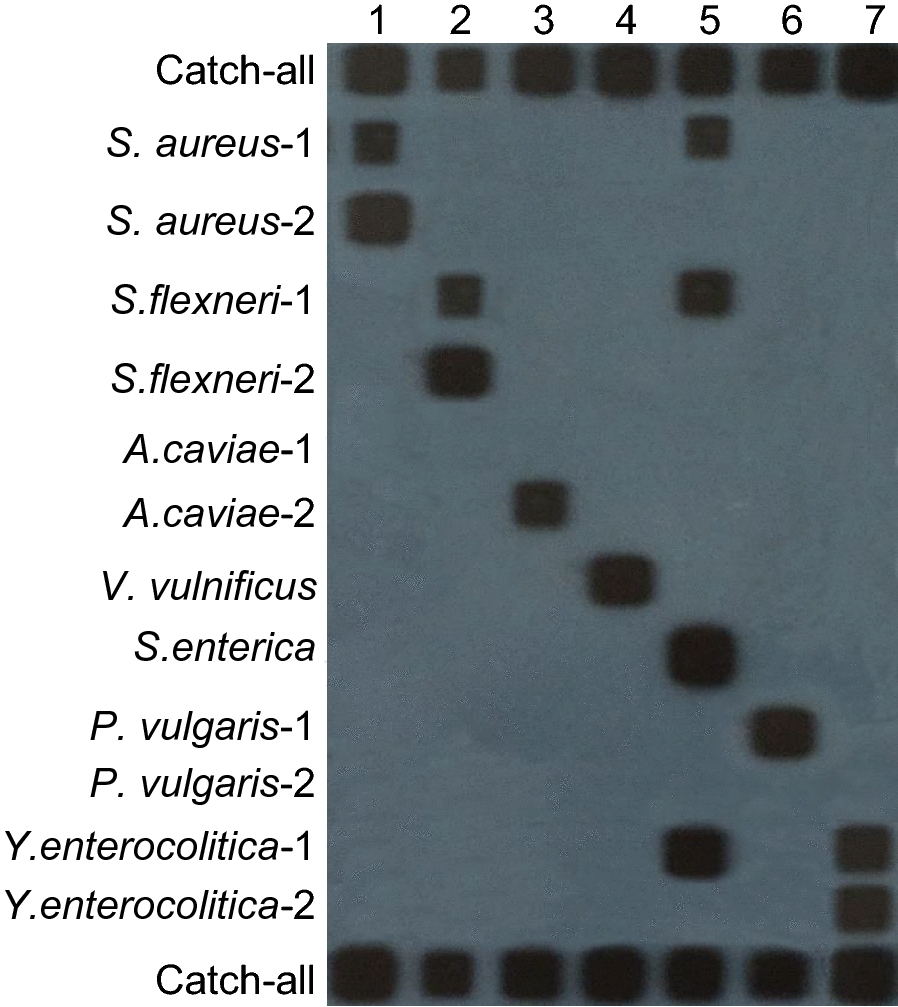
Probe selection. Oligonucleotide probes were applied in horizontal rows, and PCR products were applied in vertical lanes. Lanes 1 to 7 indicate PCR products of the 7 standard strains (*Staphylococcus aureus*, *Shigella flexneri*, *Aeromonas caviae*, *Vibrio vulnificus*, *Salmonella enterica*, *Proteus vulgaris*, and *Yersinia enterocolitica*, respectively). Rows 1 and 14 represent catch-all probes, while rows 2 to 13 correspond to *S. aureus*-1, *S. aureus*-2, *S. flexneri*-1, *S. flexneri*-2, *A. caviae*-1, *A. caviae*-2, *V. vulnificus*, *S. enterica*, *P. vulgaris*-1, *P. vulgaris*-2, *Y. enterocolitica*-1, and *Y. enterocolitica*-2 probes, respectively

The *A. caviae*-1 probe and *P. vulgaris*-2 probe did not exhibit any cross-reaction with the seven standard strains. However, *S. aureus*-1 and *S. flexneri*-1 probes simultaneously identified two bacterial species. Consequently, these five oligonucleotide probes were deemed unsuitable for PCR-RLB experiments. The final selection of oligonucleotide probes included *S. aureus*-2, *S. flexneri*-2, *A. caviae*-2, *V. vulnificus*, *S. enterica*, *P. vulgaris*-1, and *Y. enterocolitica*-2, as shown in Fig. 2.

### ***Specificity of PCR-RLB***

**Fig. 3:**
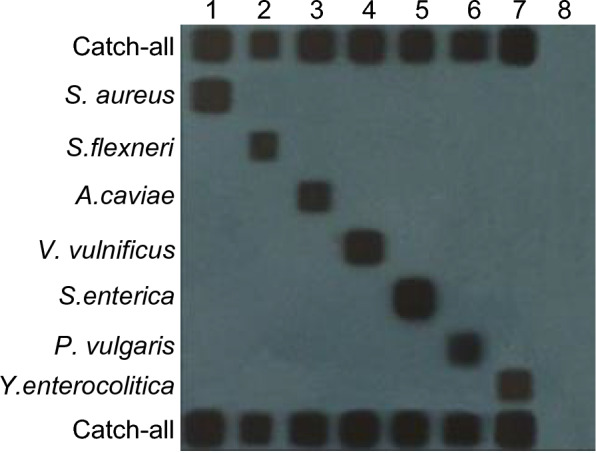
PCR-RLB specificity experiment results of seven strains. Oligonucleotide probes are represented in horizontal rows, while PCR products are shown in vertical lanes. Lanes 1 to 7 indicate PCR products of the seven standard strains (*Staphylococcus aureus*, *Shigella flexneri*, *Aeromonas caviae*, *Vibrio vulnificus*, *Salmonella enterica*, *Proteus vulgaris*, and *Yersinia enterocolitica*, respectively); 8 indicates a blank control. Rows 1 and 9 indicate catch-all; 2–8 represent *S. aureus*-2, *S. flexneri*-2, *A. caviae*-2, *V. vulnificus*, *S. enterica*, *P. vulgaris*-1, and *Y. enterocolitica*-2 probes, respectively

The PCR products, obtained from the amplification of DNAs extracted from the seven standard strains, were subjected to hybridization with probes affixed to the membrane. The resulting hybridized products displayed distinct and measurable chemiluminescent signals on the film. The specific oligonucleotide probes effectively bound to their corresponding standard strains, yielding clear chemical signals. Importantly, no cross-reaction was observed among the tested bacterial species, demonstrating the accurate identification of mixed DNAs from these diverse bacterial species (Fig. 3).

### Sensitivity of PCR-RLB

**Fig. 4:**
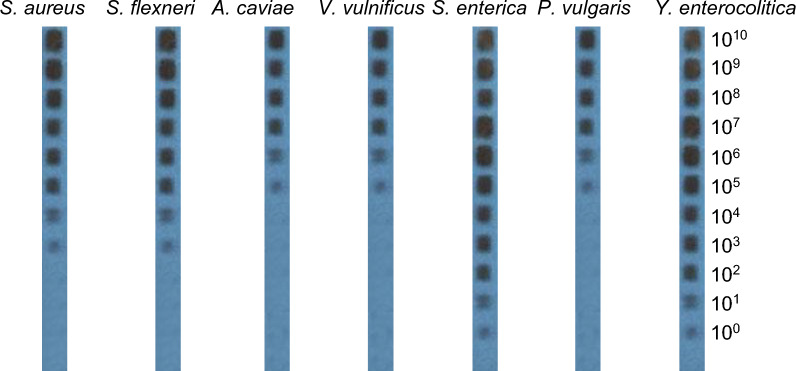
PCR-RLB sensitivity experiment results for seven strains. Oligonucleotide probes are represented in vertical lanes, and the copy numbers (copies/μl) of the serial tenfold dilutions of genomic DNA are displayed in horizontal rows 1–11, respectively

**Fig. 5:**
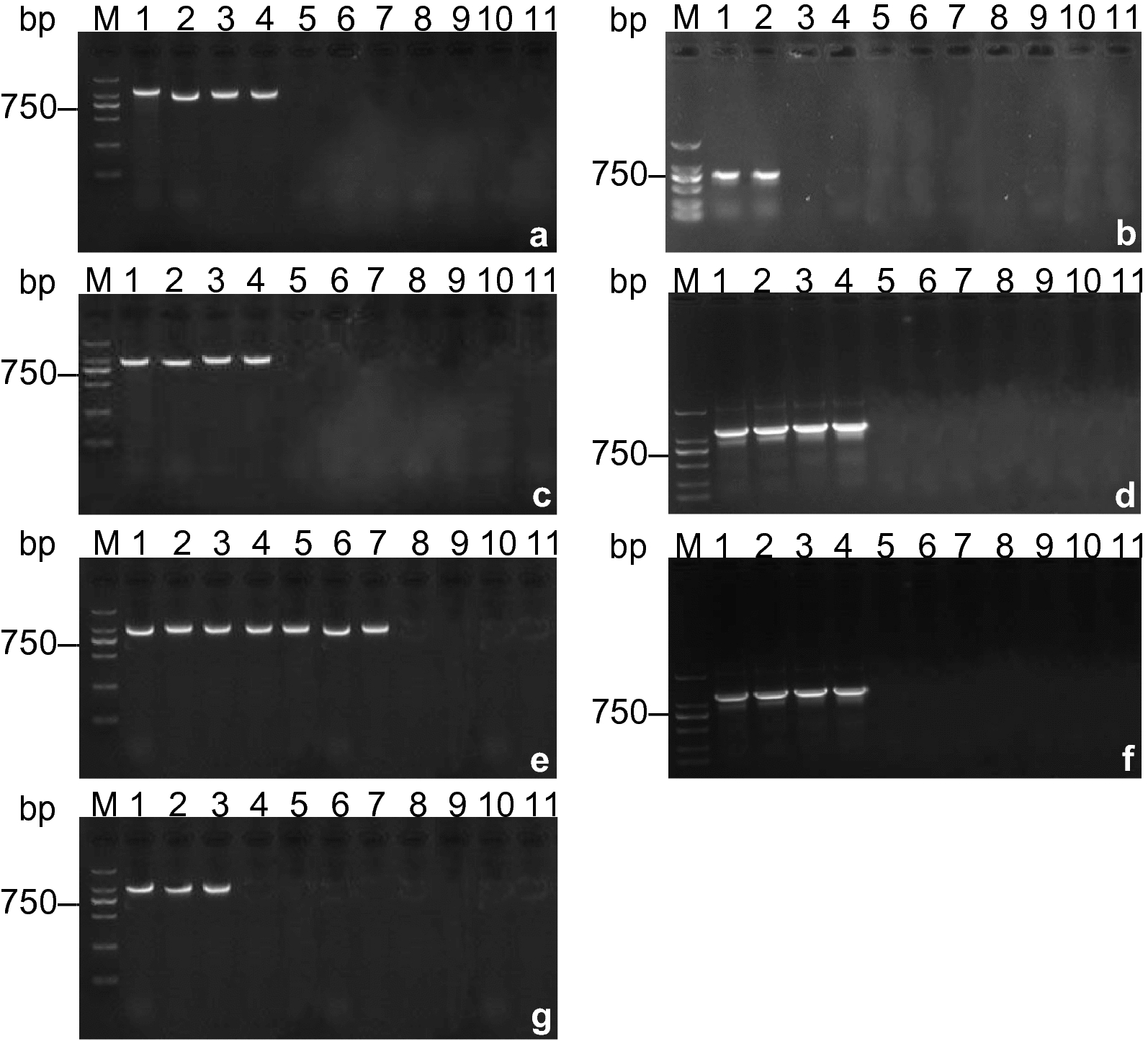
Results of PCR sensitivity for the detection of seven bacterial strains (**a**–**g**). Ten-fold serial dilutions of genomic DNA (10^10^ to 10^0^ copies/µl) were prepared using distilled water as a diluent and then amplified using PCR. Pictures a to g represent PCR results for the seven strains (*Staphylococcus aureus*, *Salmonella flexneri*, *Aeromonas caviae*, *Vibrio vulnificus*, *Salmonella enterica*, *Proteus vulgaris*, and *Yersinia enterocolitica*, respectively). Lane M contains the DL2000 DNA marker. Lanes 1 to 11 represent amplification results for the tenfold serial dilutions descending order

The PCR-RLB assay is capable of detecting bacterial strains of about 10^3^ copies/μl for *S. aureus*, 10^3^ copies/μl for *S. flexneri*, 10^5^ copies/μl for *A. caviae*, 10^5^ copies/μl for *V. vulnificus*, 10^0^ copies/μl for *S. enterica*, 10^5^ copies/μl for *P. vulgaris*, and 10^0^ copies/μl for *Y. enterocolitica* (Fig. 4). To test the ability of the developed PCR-RLB assay for detecting these seven bacterial species, a comparative evaluation with traditional PCR was conducted. The sensitivity of traditional PCR is presented in Fig. 5, with detection limits of 10^7^ copies/μl (*S. aureus*), 10^9^ copies/μl (*S. flexneri*), 10^7^ copies/μl (*A. caviae*), 10^7^ copies/μl (*V. vulnificus*), 10^4^ copies/μl (*S. enterica*), 10^7^ copies/μl (*P. vulgaris*), and 10^8^ copies/μl (*Y. enterocolitica*). The results revealed that the sensitivity of PCR-RLB was significantly higher, approximately 100 times, than that of PCR, consistent with findings in the literature [21–26].

### Simultaneous detection of seven fly-borne bacterial pathogenic from field samples by PCR-RLB

**Fig. 6:**
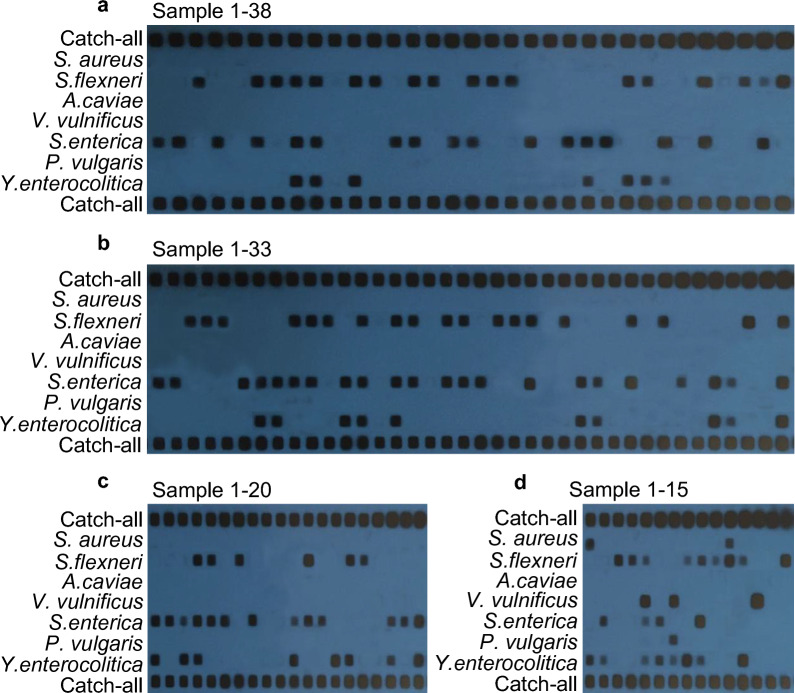
Detection of bacteria in flies from four distinct environments in Lanzhou, organized into 106 groups (comprising 10 samples per group). The y-axis represents oligonucleotide probes, the x-axis represents samples, and the detection outcomes for the 106 groups of fly samples are presented in lanes. **a** Lanes 1–38 display the detection results for 38 groups of samples from residential areas. **b** Lanes 1–33 display the detection results for 33 groups of samples from the slaughterhouse. **c** Lanes 1–20 display the detection results for 20 groups of samples from the garbage transfer station. **d** Lanes 1–38 display the detection results for 38 groups of samples from the nearby hospital

**Fig. 7:**
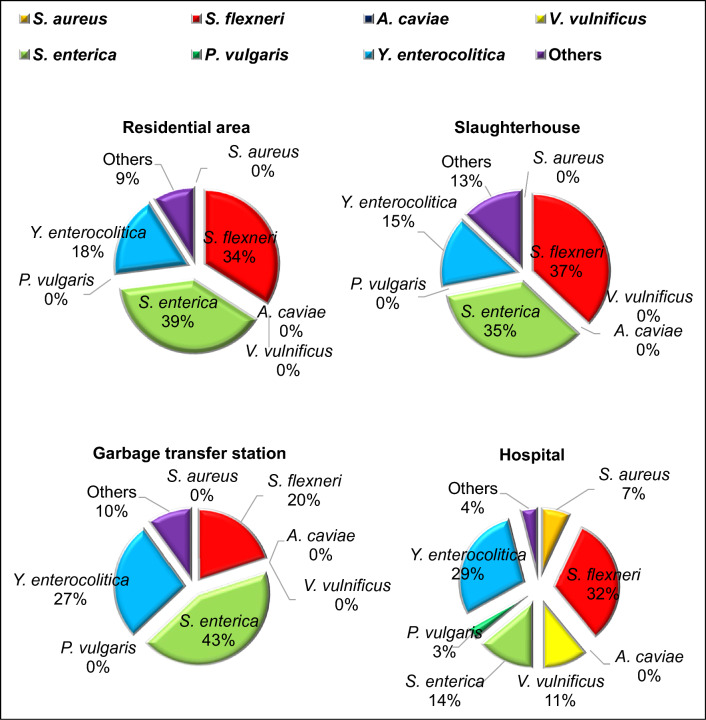
Distribution of proportions for seven pathogenic strains carried by houseflies in four different environments in Lanzhou

Simultaneous detection of seven fly-borne bacterial pathogens from field samples by PCR-RLB was conducted on a total of 1060 houseflies, grouped into 106 collections, collected from various environments in Lanzhou, China. Compared to traditional PCR, the PCR-RLB method demonstrated precise identification of different bacterial species through species-specific oligonucleotide probes. Moreover, unknown bacterial species were detectable using universal probes. The results, presented in Fig. 6, vividly illustrate the bacterial carriage status of the samples, with detailed analysis results provided in Fig. 7 and Table 4.

**Table 4:** Analysis of bacterial carrier rates for seven fly-borne bacterial pathogens

	Residential area (38 groups)	Slaughterhouse (33 groups)	Garbage transfer station (20 groups)	Hospital (15 groups)	Total (groups)	Carrier rate (%)
*Staphylococcus aureus*	0	0	0	2	2	1.23
*Shigella flexneri*	19	18	6	9	52	32.1
*Aeromonas caviae*	0	0	0	0	0	0
*Vibrio vulnificus*	0	0	0	3	3	1.85
*Salmonella enterica*	22	17	13	4	56	34.57
*Proteus vulgaris*	0	0	0	1	1	0.62
*Yersinia enterocolitica*	10	7	8	8	33	20.37
Others	5	6	3	1	15	9.26
Total	56	48	30	28	162	

The carrier rates of the seven pathogenic bacteria in samples from four different environments were as follows: 1.23% for *S. aureus*, 32.1% for *S. flexneri*, 0% for *A. caviae*, 1.85% for *V. vulnificus*, 34.57% for *S. enterica*, 0.62% for *P. vulgaris*, and 20.37% for *Y. enterocolitica*. Notably, *A. caviae* was not detected in any of the samples. The pathogen species most commonly carried by houseflies were *S. flexneri*, *S. enterica*, and *Y. enterocolitica*. Houseflies near hospitals exhibited positivity for almost all these pathogenic species, except for *A. caviae*. Residential areas and garbage transfer stations had the highest carrier rates of *Y. enterocolitica*, while slaughterhouses and areas near hospitals had the highest carrier rates of *S. flexneri*.

## Discussion

The PCR-RLB detection results emphasize the critical role of flies as vectors for intestinal infectious diseases and bacterial food poisoning. Flies collected from residential areas and slaughterhouses were found to carry a significant load of intestinal pathogens, including *S. flexneri*, *S. enterica*, and *Y. enterocolitica*. This underscores the importance of flies as carriers of intestinal infectious diseases, as these pathogens can cause symptoms such as diarrhea and vomiting. Flies carrying these pathogenic bacteria can contaminate food, utensils, everyday items, and their surroundings, increasing the risk of intestinal infectious diseases and bacterial food poisoning through contact or ingestion. Flies near hospitals were found to not only carry common intestinal pathogens but also *S. aureus* and *Vibrio vulnificus*. Hence, hospitals should take effective measures to control fly populations and strengthen the prevention and control of fly-borne bacterial diseases. These findings underscore the importance of improving urban environments and curbing the transmission of disease vectors to effectively control the spread of insect-borne diseases.

In this study, we developed a sensitive, reliable, and rapid method for the simultaneous detection of multiple fly-borne bacterial pathogens. The species-specific probes designed for PCR-RLB showed high specificity, only hybridizing with amplified DNA from the corresponding species. Additionally, the membrane-bound oligonucleotide species-specific probes used in PCR-RLB detection technology can be easily reused for bacterial detection after washing with 0.5 M EDTA, significantly enhancing detection efficiency.

Sensitivity tests were conducted on PCR amplification products with different copy numbers, prepared by continuous tenfold dilution. The results demonstrated that the sensitivity of PCR-RLB was substantially higher than that of individual PCR (approximately 100 times). While establishing PCR-RLB requires special nylon membranes (Biodyne) and access to professional laboratories and technical expertise, the actual detection process is straightforward, requiring only a membrane and a water bath to analyze multiple samples. The membrane can be reused 4–6 times, resulting in significant cost savings and making this method applicable in traditional laboratories. A limitation of the method was the long length of the PCR amplification, which affects sensitivity.

## Conclusions

This newly established detection method was employed to collect data on bacteria carried by houseflies randomly collected from four different environments in Lanzhou, China. This preliminary exploration sheds light on how different urban environments impact fly-borne bacteria. The results indicate that this rapid detection method for intestinal infectious bacteria carried by flies holds potential clinical application value and represents a crucial measure for preventing and controlling infectious diseases.

## Data Availability

The original contributions presented in the study are included in the article/Supplementary material; further inquiries can be directed to the corresponding authors.

## Abbreviations

PCR-ELISA: Polymerase chain reaction-enzyme linked immunosorbent assay
qPCR: Real-time PCR
PCR-RLB: PCR-based reverse line blot
NCBI: National Center for Biotechnology Information
BLASTn: Basic Local Alignment Search Tool

## References

[1] Onwugamba FC, Fitzgerald JR, Rochon K, Guardabassi L, Alabi A, Kühne S (2018). The role of 'filth flies' in the spread of antimicrobial resistance. Travel Med Infect Dis.

[2] Galuppi R, Bonoli C, Aureli S, Cassini R, Marcer F, Foley JE (2012). Comparison of diagnostic methods to detect piroplasms in asymptomatic cattle. Vet Parasitol.

[3] Yap KL, Kalpana M, Lee HL (2008). Wings of the common house fly (*Musca domestica* L.): Importance in mechanical transmission of Vibrio cholerae. Trop Biomed.

[4] Liu Y, Chen Y, Wang N, Qin H, Zhang L, Zhang S (2023). The global prevalence of parasites in non-biting flies as vectors: a systematic review and meta-analysis. Parasit Vectors.

[5] Tong SYC, Davis JS, Eichenberger E, Holland TL, Fowler VG (2015). *Staphylococcus aureus* infections: epidemiology, pathophysiology, clinical manifestations, and management. Clin Microbiol Rev.

[6] Wasala L, Talley JL, Desilva U, Fletcher J, Wayadande A (2013). Transfer of *Escherichia coli* O157:H7 to spinach by house flies, *Musca domestica* (Diptera: Muscidae). Phytopathology.

[7] Zhang J, Wang J, Chen L, Yassin AK, Kelly P, Butaye P (2017). Housefly (*Musca domestica*) and blow fly (*Protophormia terraenovae*) as vectors of bacteria carrying colistin resistance genes. Appl Environ Microbiol.

[8] Khamesipour F, Lankarani KB, Honarvar B, Kwenti TE (2018). A systematic review of human pathogens carried by the housefly (*Musca domestica* L.). BMC Public Health.

[9] Förster M, Klimpel S, Sievert K (2009). The house fly (*Musca domestica*) as a potential vector of metazoan parasites caught in a pig-pen in Germany. Vet Parasitol.

[10] Gerry AC (2020). Review of methods to monitor house fly (*Musca domestica*) abundance and activity. J Econ Entomol.

[11] Chaiwong T, Srivoramas T, Sueabsamran P, Sukontason K, Sanford MR, Sukontason KL (2014). The blow fly, *Chrysomya megacephala*, and the house fly, *Musca domestica*, as mechanical vectors of pathogenic bacteria in Northeast Thailand. Trop Biomed.

[12] Hogsette JA (2021). Factors affecting numbers of house flies (Diptera: *Muscidae*) captured by ultraviolet light traps in a large retail supermarket. J Econ Entomol.

[13] Gill C, Bahrndorff S, Lowenberger C (2017). *Campylobacter jejuni* in *Musca domestica*: An examination of survival and transmission potential in light of the innate immune responses of the house flies. Insect science.

[14] Prosdocimi EM, Mapelli F, Gonella E, Borin S, Crotti E (2015). Microbial ecology-based methods to characterize the bacterial communities of non-model insects. J Microbiol Methods.

[15] Sue MJ, Yeap SK, Omar AR, Tan SW (2014). Application of PCR-ELISA in molecular diagnosis. Biomed Res Int.

[16] Pansri P, Svensmark B, Liu G, Thamsborg SM, Kudirkiene E, Nielsen HV (2022). Evaluation of a novel multiplex qPCR method for rapid detection and quantification of pathogens associated with calf diarrhoea. J Appl Microbiol.

[17] Kaufhold A, Podbielski A, Baumgarten G, Blokpoel M, Top J, Schouls L (1994). Rapid typing of group A *streptococci* by the use of DNA amplification and non-radioactive allele-specific oligonucleotide probes. FEMS Microbiol Lett.

[18] O'Sullivan MV, Zhou F, Sintchenko V, Kong F, Gilbert GL (2011). Multiplex PCR and reverse line blot hybridization assay (mPCR/RLB). J Vis Exp.

[19] Nijhof AM, Pillay V, Steyl J, Prozesky L, Stoltsz WH, Lawrence JA (2005). Molecular characterization of *Theileria* species associated with mortality in four species of African antelopes. J Clin Microbiol.

[20] Allsopp MT, Hattingh CM, Vogel SW, Allsopp BA (1998). Comparative evaluation of 16S, map1 and pCS20 probes for the detection of *Cowdria* and *Ehrlichia* species in ticks. Ann N Y Acad Sci.

[21] Graczyk TK, Fayer R, Knight R, Mhangami-Ruwende B, Trout JM, Da Silva AJ (2000). Mechanical transport and transmission of *Cryptosporidium parvum* oocysts by wild filth flies. Am J Trop Med Hyg.

[22] Christova I, Van De Pol J, Yazar S, Velo E, Schouls L (2003). Identification of *Borrelia burgdorferi sensu lato*, *Anaplasma* and *Ehrlichia* species, and spotted fever group *Rickettsiae* in ticks from Southeastern Europe. Eur J Clin Microbiol Infect Dis.

[23] Nijhof AM, Penzhorn BL, Lynen G, Mollel JO, Morkel P, Bekker CP (2003). *Babesia bicornis sp. nov.* and *Theileria bicornis sp. nov*.: tick-borne parasites associated with mortality in the black rhinoceros (*Diceros bicornis*). J Clin Microbiol.

[24] Oura CA, Bishop RP, Wampande EM, Lubega GW, Tait A (2004). Application of a reverse line blot assay to the study of haemoparasites in cattle in Uganda. Int J Parasitol.

[25] Ranka R, Bormane A, Salmina K, Baumanis V (2004). Identification of three clinically relevant *Borrelia burgdorferi sensu lato* genospecies by PCR-restriction fragment length polymorphism analysis of 16S–23S ribosomal DNA spacer amplicons. J Clin Microbiol.

[26] Wang HY, Kim H, Kim S, Bang H, Kim DK, Lee H (2015). Evaluation of PCR-reverse blot hybridization assay for the differentiation and identification of *Mycobacterium* species in liquid cultures. J Appl Microbiol.

